# Genome Sequencing of Idiopathic Speech Delay

**DOI:** 10.1155/2024/9692863

**Published:** 2024-03-28

**Authors:** Else Eising, Arianna Vino, Heather L. Mabie, Thomas F. Campbell, Lawrence D. Shriberg, Simon E. Fisher

**Affiliations:** ^1^Language and Genetics Department, Max Planck Institute for Psycholinguistics, 6525 XD Nijmegen, Netherlands; ^2^Waisman Center, University of Wisconsin-Madison, Madison, WI 53705, USA; ^3^School of Behavioral and Brain Sciences, Callier Center for Communication Disorders, University of Texas at Dallas, Dallas, USA; ^4^Donders Institute for Brain, Cognition and Behaviour, Radboud University, 6525 EN Nijmegen, Netherlands

## Abstract

Genetic investigations of people with speech and language disorders can provide windows into key aspects of human biology. Most genomic research into impaired speech development has so far focused on childhood apraxia of speech (CAS), a rare neurodevelopmental disorder characterized by difficulties with coordinating rapid fine motor sequences that underlie proficient speech. In 2001, pathogenic variants of *FOXP2* provided the first molecular genetic accounts of CAS aetiology. Since then, disruptions in several other genes have been implicated in CAS, with a substantial proportion of cases being explained by high-penetrance variants. However, the genetic architecture underlying other speech-related disorders remains less well understood. Thus, in the present study, we used systematic DNA sequencing methods to investigate idiopathic speech delay, as characterized by delayed speech development in the absence of a motor speech diagnosis (such as CAS), a language/reading disorder, or intellectual disability. We performed genome sequencing in a cohort of 23 children with a rigorous diagnosis of idiopathic speech delay. For roughly half of the sample (ten probands), sufficient DNA was also available for genome sequencing in both parents, allowing discovery of *de novo* variants. In the thirteen singleton probands, we focused on identifying loss-of-function and likely damaging missense variants in genes intolerant to such mutations. We found that one speech delay proband carried a pathogenic frameshift deletion in *SETD1A*, a gene previously implicated in a broader variable monogenic syndrome characterized by global developmental problems including delayed speech and/or language development, mild intellectual disability, facial dysmorphisms, and behavioural and psychiatric symptoms. Of note, pathogenic *SETD1A* variants have been independently reported in children with CAS in two separate studies. In other probands in our speech delay cohort, likely pathogenic missense variants were identified affecting highly conserved amino acids in key functional domains of *SPTBN1* and *ARF3*. Overall, this study expands the phenotype spectrum associated with pathogenic *SETD1A* variants, to also include idiopathic speech delay without CAS or intellectual disability, and suggests additional novel potential candidate genes that may harbour high-penetrance variants that can disrupt speech development.

## 1. Introduction

Most children learn to speak within the first few years of life without exceptional effort or formal training. Typically, exposure to linguistic input in their environment is enough for children to develop the skills needed to understand others and to produce speech. However, for a subset of children, the path to intelligible speech is not so straightforward. Children with delayed speech development take longer to become proficient in this skill, demonstrated by (for example) age-inappropriate speech sound deletions and/or substitutions [1]. Sometimes this occurs in the context of broader developmental problems such as intellectual disability, deafness, autism, or neurological damage, but in the case of idiopathic speech delay, such diagnoses are absent. Speech delay may be considered as a subtype of a more general category of speech sound disorders (SSD) that also includes speech errors like lisps and motor speech disorders like childhood apraxia of speech (CAS) [2]. Children with persistent speech delay often experience difficulties in other domains as well and may present with reading difficulties [3] and/or developmental language disorder [4]. However, for children with speech delay who do not experience other language-related problems or intellectual disability, intelligibility usually develops to a level that is consistent with their intellectual abilities within two to three years following diagnosis [5].

Several lines of evidence suggest that speech- and language-related skills and their associated disorders have strong genetic underpinnings [6]. Most of the heritable variation in speech- and language-related traits is likely to involve an interplay of many genetic factors with small effect sizes. Genome-wide association analyses have identified the first common genetic variants associated with reading and dyslexia and could explain 8 to 26% of variability in reading, spelling, phoneme awareness, nonword repetition, dyslexia, and vocabulary in infants and toddlers by common variants (so-called SNP heritability) [7–9]. However, there are rare cases documented where a speech and language disorder occurs in a monogenic form. The most studied examples thus far relate to the disorder CAS, for which the burden of rare high-penetrance DNA variants appears relatively high. The first findings in this area date back to 2001, when mutation of *FOXP2* was identified as the cause of CAS in fifteen affected (but no unaffected) relatives in a large multigenerational family (MIM: 602081) [10]. Following this, additional inherited and *de novo* disruptive *FOXP2* variants have been implicated in speech/language deficits (with CAS as the most prominent phenotype) in multiple independent families and probands [11], but these still explain only a small proportion of cases overall [12]. In recent years, systematic exome/genome sequencing screens of CAS cohorts have begun to identify potential high-penetrance pathogenic variants in genes beyond *FOXP2* [6, 13–15]. Most of the genetic loci highlighted by such CAS screens have also been implicated in heterogeneous neurodevelopmental disorders, in which speech problems may occur in the context of a more pervasive syndrome, with notable examples including *CHD3* [16] (MIM: 618205), *SETD1A* [17] (MIM: 619056), *WDR5* [18], and *SETBP1* [19] (MIM: 616078). It is not yet well understood why variants in the same gene yield more selective issues with speech in some affected individuals but cause broader syndromes involving multiple aspects of the brain and behaviour/cognition in others [20]. Moreover, systematic characterization of speech phenotypes is seldom carried out when collecting clinical information of children with monogenic neurodevelopmental syndromes, and for some established disorders, problems in this area may be more central than initially thought. This is, for example, evident from a recent assessment of the communication profiles of individuals with KAT6A syndrome (MIM: 616268), showing that severe communication difficulties are a core feature of the disorder [21]. More thorough phenotypic characterizations of existing syndromes, as well as the identification of additional pathogenic DNA variants in different loci, are required to obtain a better picture of how genetic risk factors may derail speech development.

In contrast to CAS, the genetic underpinnings of idiopathic speech delay have yet to be investigated with next-generation DNA sequencing methods. Identifying whether high-penetrance genetic variants can lead to monogenic subtypes of speech delay, and characterizing the relevant genetic loci, will enhance understanding of pathways underlying speech (dys)function. In addition, by only including cases of idiopathic speech delay, in the absence of a motor speech disorder or intellectual disability, we can study whether there are genetic overlaps between speech delay and broader neurodevelopmental disorders, even when some cases do not show phenotypic overlap, as demonstrated for CAS. Here, we used genome sequencing of 23 children with a rigorous diagnosis of idiopathic speech delay, with neither a motor speech disorder nor intellectual disability, to search for potentially pathogenic single-nucleotide variants, as well as small and large insertions or deletions. For ten probands, we had the possibility to also sequence the genomes of both parents, allowing us to discover *de novo* variants. We used strict filtering of the variants uncovered, which resulted in the identification of a pathogenic or likely pathogenic variant in three cases from the cohort.

## 2. Material and Methods

### 2.1. Participants

Probands were selected from children participating in a diagnostic classification and genetic study of children with speech sound disorders conducted at the Callier Center for Communication Disorders, University of Texas at Dallas. Protocols for participant recruitment, speech-language assessment, and saliva collection for the genetic study were approved by Institutional Review Boards at the University of Wisconsin-Madison and the University of Texas at Dallas. All participants provided informed consent. Potential study participants were identified by referring certified speech-language pathologists. A telephone screening was conducted by research personnel supported by a parent or caregiver to identify potential study participants who met the following inclusionary criteria: moderate-to-severe speech delay; 3 to 8 years of age; no intellectual, structural, hearing, neurological, or affective disorder; and from a home in which English was the only or primary spoken language.

A total of 67 participants for the diagnostic classification study were assessed using the Madison Speech Assessment Protocol [2], a two-hour battery of 25 measures that includes 15 speech tests and tasks scored using auditory-perceptual and acoustic methods. The Speech Disorder Classification System (SDCS) [2, 22] was used to cross-classify the speech and motor speech status of each participant. All participants met SDCS criteria for idiopathic speech delay. Additionally, according to this scheme, their motor speech status was classified into one of the five classifications (no motor speech disorder), or one of four types of motor speech disorder (speech motor delay, childhood dysarthria, childhood apraxia of speech, or childhood dysarthria and childhood apraxia of speech). In addition, the Detroit Test of Learning Aptitude-Primary (DTLA; 3rd Edition) [23] was used to assess the cognitive ability of the participants. Saliva samples for DNA isolation were collected from probands and some nuclear family members using Oragene saliva collection kits (DNA OG-500 kit; DNA Genotek Inc., Kanata, Ontario, Canada).

Probands classified as having concurrent idiopathic speech delay and no motor speech disorder at assessment were considered further for the present genome sequencing analysis. Cases with more severe speech delay were selected, based on scoring at least two standard deviations below the mean on one of the two speech competence markers: percentage vowels correct and percentage consonants correct [2]. In addition, probands were excluded if their general ability standardized score was below 80, or substantially lower than the estimated IQ of at least one of their parents based on the parent's education level. For two cases, the DTLA had not been administered, so their general ability was not scored.

DNA from parents was included when it was available from both parents and no verbal trait disorder was reported in either parent. After applying these criteria, a total of 23 probands were included in the genome sequencing analyses. A trio sequencing strategy was applied for ten probands, who had DNA available from both parents. The other 13 probands were included with a singleton strategy. Phenotype descriptions of these probands are summarized in Table S1.

### 2.2. Genome Sequencing and Variant Calling

Genome sequencing was performed by Novogene (Hong Kong). Paired-end sequencing was carried out on the Illumina HiSeq Xten platform, with reads of 150 base pairs. The data comprised on average 740 million reads per sample (range 599 to 1067 million) and an average sequencing depth of 33.1 times (range 19.2 to 47.8). The sequencing data were mapped onto the human reference genome (GRCh37) using the software Burrows-Wheeler Aligner (BWA) [24] and then processed according to Genome Analysis Toolkit Software Best Practices (GATK v4.0.1.1) [25]. First, PCR-duplicated reads were marked using Picard, and BAM files were sorted using SAMtools (v1.3.1) [26]. Genetic variants in the sequence data were called using HaplotypeCaller, consolidated using GenomicsDBImport, and merged together using the GenotypeGVCFs, three tools of GATK [25]. Lastly, we performed Variant Quality Score Recalibration (VQSR) on the genome sequence data and excluded variants with a VQSR score over 99%. All variants discussed in the manuscript were independently validated using Sanger sequencing. When DNA was available, Sanger sequencing was also used to study the presence/absence of the variant in the parents of the proband, independent of whether genome sequence data were available from the parents.

### 2.3. Structural Variant Calling

Structural variants were called using BreakDancer (v1.1.2) [27] and BIC-seq2 (BIC-seq-norm v0.2.4 and BIC-seq-seq v0.7.2) [28]. BreakDancer calls structural variants based on the alignment of read pairs and was run on the pooled dataset using standard settings. BIC-seq2 detects deletions and duplications based on the comparison of read depth between two samples. Probands in the proband-parent trios were compared to both parents to identify *de novo* deletions and duplications. Singleton probands were compared to two unrelated parents. Unique sequencing reads with a quality score ≥ 20 were extracted using SAMtools, after which BIC-seq2 was used to normalize the data and identify structural variants. Lambda was set to 0.5 for more lenient detection of deletions and duplications. Structural variants were considered if they were detected by both tools (maximum twofold size difference and maximal distance of 10 kb between predicted start or end sites), were not detected in any of the parents by BreakDancer, and were located in one or more exons of a protein-coding gene.

### 2.4. Variant Annotation and Filtering

Variants were annotated with ANNOVAR [29] (version 2017-07-17) and subsequently filtered. Only exonic variants in protein-coding genes were included. Variants located in known regions of genomic duplications were removed, as were variants with three or fewer reads supporting either allele. For the proband-parent trios, *de novo* variants were identified as those variants present in the proband but not in the parents. For the single probands, variants not present in either of the parents of any of the trios were selected. Variants were further filtered based on minor allele frequency (MAF), gene intolerance, predicted functional impact of the variant, and expression of the gene in developing brain, according to thresholds outlined below. Strict filtering criteria were used to prevent false-positive findings, even though they might also filter out true causal variants.

Only variants with MAF < 3.2 × 10^−4^ were considered. This threshold was based on a statistical framework that takes into account disease prevalence, genetic heterogeneity, and penetrance [30]. We used a population frequency of 2.9%, as the prevalence of speech delay in 4-8-year-olds has been estimated at 3.6% [4, 31, 32], of which 82% have no motor speech disorder [22]. We used lenient values for heterogeneity (0.02; i.e., no single variant causes more than 2% of cases) and penetrance (90%), since these values are unknown for speech delay. The Genome Aggregation Database [33] (gnomAD, v2.1.1) and Known VARiants database [34] (Kaviar, version 2015-09-23) were used as reference.

For genes with pLoF variants and structural variants, gene intolerance was based on the probability of being loss-of-function intolerant (pLI) score [35]. For genes with missense variants, gene intolerance was based on the *Z*-score for missense constraint (MIS_Z) [35], pLI score, and the local tolerance score from Metadome [36]. The pLI and MIS_Z scores are calculated from the ratio between the number of observed variants and the number of variants expected based on the DNA sequence of the gene. The local tolerance score from Metadome is calculated as a missense over synonymous variant count ratio, in a sliding window manner, to provide a per-position indication of regional tolerance to missense variation. All scores are based on the sequencing data in gnomAD. Genes with pLI > 0.9 were considered intolerant to pLoF variants and structural variants. For missense variants, genes with MIS_Z > 2.5 or pLI > 0.9 and amino acids with a local tolerance score indicating an intolerant locus were considered intolerant.

The impact of pLoF variants was predicted based on their location in the gene. Splicing variants were included only if they affected the main acceptor and donor sites. Frameshift and stop-gain variants were excluded when located within 50 base pairs from the end of the transcript, unless they affected a protein domain. The impact of missense variants was predicted based on ratings of base-specific evolutionary constraint using Genomic Evolutionary Rate Profiling (GERP++) and three algorithms that predict functional effects of human SNPs: rare exome variant ensemble learner (REVEL) [37], Polymorphism Phenotyping-2 (PolyPhen-2) [38], and Sorting Tolerant From Intolerant (SIFT) [39]. Missense variants with GERP > 2, REVEL > 0.5, and PolyPhen and/or SIFT indicating a (possibly) damaging effect were considered to have high impact.

Speech delay is a neurodevelopmental disorder; therefore, only variants in transcripts that are expressed in the human brain were included. Expression levels of the genes and exons carrying the variants were assessed in the developmental human brain RNA-sequencing dataset of Brainspan [40] and the adult human brain gene expression data in GTEx [41].

### 2.5. Variant Interpretation

Phenotypes previously associated with similar variants (either pLoF or missense) occurring in the same gene were collected using searches in PubMed, the Online Mendelian Inheritance in Man (OMIM) database, denovo-db (v1.6.1) [42], and VariCarta [43] (assessed in May 2023). For variants in genes previously associated with a neurodevelopmental disorder, available interpretations of pathogenicity were obtained from ClinVar [44]. Remaining variants were interpreted according to a five-tier system of classification for variants of Mendelian disorders into (1) pathogenic, (2) likely pathogenic, (3) uncertain significance, (4) likely benign, and (5) benign variants [45]. This approach for interpreting variants has limited power to detect new gene-disease associations, as variants in known causal disease genes require less additional proof before being classified as (likely) pathogenic than variants in genes that not yet have been described as causal for a neurodevelopmental disorder. Moreover, it is unlikely to identify causal genes through recurrence (i.e., identifying multiple mutations in the same gene), an approach which would not have be affected by known gene-disease associations, due to the limited sample size. Effects of pLoF variants on nonsense mediated mRNA decay was studied using NMDEscPredictor [46].

## 3. Results

**Figure 1:**
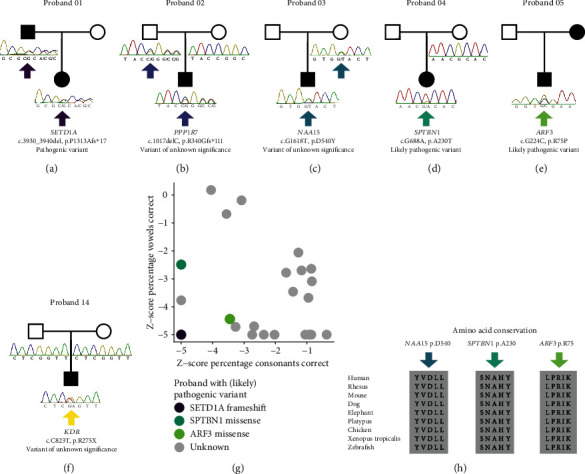
Inheritance pattern of variants in six probands, the severity of speech delay, and conservation of the missense variants. (a–f) Sanger validation and segregation analysis of variants. Sanger sequencing traces are shown for all individuals with DNA available. Pedigrees show circles/squares filled black for probands, all of whom had speech delay, or for parents with self-reported speech and/or language difficulties. Circles/squares filled white represent parents without self-reported speech and/or language difficulties. (a) Frameshift variant in *SETD1A* in proband 01 that was inherited from the father, who self-reported problems with speech, reading, learning, and cognition. (b) Frameshift variant in *PPP1R7* in proband 02 that was inherited from the father, who did not self-report any speech-related problems. (c) Missense variant in *NAA15* in proband 03, inherited from the mother, who did not self-report any speech/language difficulties. (d) Missense variant in *SPTBN1* in proband 04. The variant was not present in the mother. (e) Missense variant in *ARF3* in proband 05. (f) *De novo* stop-gain pLoF variant in *KDR* in proband 06. (g) Speech competence of the speech delay cohort, based on percentage consonants and vowels correct. The probands who carry a variant classified as (likely) pathogenic are highlighted. (h) Amino acid evolutionary conservation of the relevant parts of proteins encoded by *NAA15, SPTBN1*, and *ARF3*. The arrows point to the amino acids affected by missense variants, each of which is highly conserved across diverse vertebrate species.

Genome sequencing was used to screen 23 probands with speech delay for potential pathogenic variants that may explain their speech phenotype. We were able to take advantage of a proband-parent trio design for ten of the probands, by sequencing the DNA of both unaffected parents and searching for *de novo* variants. For the other thirteen probands, either DNA was not available in large enough quantity for both parents or a parent reported problems with speech or related issues. For these thirteen probands, we focused on rare pLoF variants and missense variants predicted to be damaging in genes intolerant to such mutations. A total of five rare pLoF variants were identified in intolerant genes (pLi > 0.9; Table 1). One pLoF variant was identified in proband 01 in *SETD1A* (p.P1313Afs^∗^17), a gene encoding a histone methyl transferase. Sanger sequencing of this gene in the father, for whom DNA was also available, showed that the *SETD1A* frameshift was inherited from him. This father self-reported problems with speech, reading, learning, and cognition but was not further assessed, and so systematic data on the nature of his speech difficulties were not available (Figure 1(a)). The variant is predicted to lead to nonsense-mediated decay and thus *SETD1A* haploinsufficiency. *SETD1A* haploinsufficiency is known to cause a Mendelian disorder characterized by global developmental delay including delayed speech and/or language development, mild intellectual disability, subtle facial dysmorphisms, and behavioural and psychiatric symptoms (MIM: 619056) [17, 47]. The *SETD1A* frameshift was therefore classified as pathogenic. Of note, proband 01 does not have symptoms indicative of developmental delay or a psychiatric disorder and therefore presents with a mild phenotype compared to others with *SETD1A* haploinsufficiency. Yet, this proband represents one of the most affected of the speech delay cohort based on two speech competence indices (percentage consonants correct and percentage vowels correct) and the Goldman-Fristoe Test of Articulation [48] (Figure 1(g) and Supplemental Table 1). Remarkably, in prior genome sequencing efforts of modest sized samples, *de novo* pLoF variants in *SETD1A* have been independently identified twice in children with CAS: one without any symptoms indicating developmental delay or psychiatric problems [13] and one with a borderline low IQ of 79 [14]. These prior findings are in line with the current results, indicating that *SETD1A* haploinsufficiency can cause a mild disorder mainly affecting speech.

The other four pLoF variants were located in genes not previously associated with a neurodevelopmental disorder according to prior literature. Proband 02 carries a frameshift variant in *PPP1R7*. A *de novo* pLoF variant was previously found in *PPP1R7* in a proband with autism spectrum disorder who was part of a large cohort of trios [49], but in that investigation, no enrichment of *de novo* variants was identified that could point towards a causal role of the gene. In our study, DNA was available from both parents for Sanger sequencing, which revealed that the *PPP1R7* frameshift variant was inherited from the father who did not report speech-related problems (Figure 1(b)). Thus, taking all evidence together, we classified this as a variant of unknown significance. Proband 04 has a pLoF variant in *TCERG1*, which is classified as variant of unknown significance because the proband also carries a likely pathogenic missense variant in *SPTBN1* (Table 2). Proband 06 carries two pLoF variants in intolerant genes *RIPOR1* and *TOP2A.* Since neither gene has yet been associated with a neurodevelopmental disorder, both were classified as variants of unknown significance.

**Figure 2:**
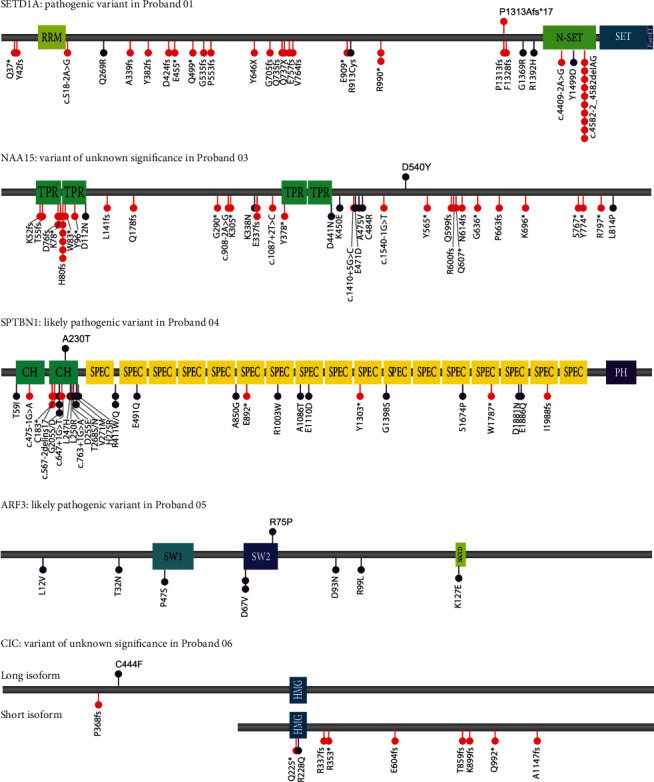
Locations of identified variants and an overview of published variants in four genes. Variants identified in this study are visualized above linear protein schematics; the variants previously published as causal for a monogenic neurodevelopmental disorders are visualized below for SETD1A [13, 14, 17, 47], NAA15 [53–55], SPTBN1 [50, 51], and ARF3 [52, 56] and the short and long isoform of CIC [57–60]. Missense variants are indicated in purple and pLoF variants in red. Protein domains are represented with coloured squares: RRM: RNA recognition motif; N-SET: COMPASS (complex proteins associated with Set1p) component N; NKXD: NKXD motif; SET: Su(var)3-9, enhancer-of-zeste, trithorax domain; Post-SET: cysteine-rich motif following a subset of SET domains; SW: switch domain; TPR: tetratricopeptide repeat; CH: calponin homology domain; SPEC: spectrin repeats; PH: Pleckstrin homology domain; HMG: high-mobility group box.

We also studied missense variants in the genomes of the thirteen probands without DNA available from both parents. After strict filtering for MAF, gene intolerance, predicted impact of the variant, and expression of the gene in the brain, a total of 18 missense variants remained (Table 2). Two missense variants were classified as likely pathogenic: p.A230T in *SPTBN1* in proband 04 and p.R75P in *ARF3* in proband 05 (Figures 1(d) and 1(e)). *SPTBN1* was recently implicated in a neurodevelopmental disorder characterized by intellectual disability, language and motor delays, autistic features, and seizures (MIM: 619475) [50, 51]. Causal missense variants that have been previously identified in *SPTBN1* mostly cluster in the second calponin homology domain [50], which is where the p.A230T variant in proband 04 is located (Figure 2). Heterozygous missense variants in *ARF3* have been associated with a developmental disorder affecting the central nervous system and skeletal system, with variable expressivity [52]. The p.R75 amino acid is located in the second switch domain (Figure 2), which is important for GTP/GMP binding and protein interactions. The amino acids affected by the missense variants in *SPTBN1* and *ARF3* are highly conserved in distantly related vertebrate species (Figure 1(h)). Compared to individuals previously described with *SPTBN1*- and *ARF3*-related disorders, probands 04 and 05 present a very mild neurodevelopmental phenotype with only speech delay. But compared to the remainder of the speech delay cohort that we screened in the current study, the speech of both probands can both be considered as highly affected (Figure 1(g) and Supplemental Table 1).

Seven other missense variants from the current cohort were identified in genes previously associated with a heterozygous neurodevelopmental disorder: *NAA15* (p.D540Y) in proband 03, *RELN* (p.G3368R) in proband 05, *HCN1* (p.G74_E75insGGG) in proband 08, *CIC* (p.C444F) in proband 06, *STXBP1* (R100W) in proband 09, and *KIF1B* (p.Y96C) in proband 02. The *NAA15* variant in 03 was also present in the mother (Figure 1(c)), who did not self-report any speech, language, or cognitive problems, but noted that her father (the grandfather of proband 03) had hearing, reading, and learning problems. PLoF variants [53] and missense variants [54] in *NAA15* have been linked to a neurodevelopmental disorder with variable levels of intellectual disability, delayed speech and motor milestones, and autism spectrum disorder (MIM: 617787). The mutated amino acid is a residue with high evolutionary conservation (Figure 1(h)). Yet, missense variants described as pathogenic or likely pathogenic are mainly located in a small region of the protein between amino acids 450 and 484 (Figure 2). Given the distance between the p.D540Y variant and this region of the protein, the variant identified in our cohort was classified as a variant of unknown significance. Missense and pLoF variants in *STXBP1* have been associated with a disorder characterized by neurodevelopmental delay, seizures, and delayed speech and language development (MIM: 612164) [67]. However, the presence of the p.R100W variant in gnomAD, conflicting interpretations in ClinVar, and its location outside hotspots with recurrent pathogenic variants [67] led it to be classified as a variant of unknown significance. Variants in *CIC* have been associated with a neurodevelopmental disorder characterized by intellectual disability, autism, and ADHD [58, 60] (MIM: 617600; Figure 2), with several pLoF variants and one missense variant in the HMG-box domain described. Because the missense variant in *CIC* identified here is not located in the HMG-box domain, it was classified as variant of unknown significance. The variants in *HCN1* and *RELN* were also classified as variants of uncertain significance and likely benign in ClinVar. Lastly, the phenotype associated with pathogenic variants of *KIF1B*, peripheral sensorimotor neuropathy named Charcot-Marie-Tooth disease type 2A1 (MIM: 118210), is highly dissimilar to speech delay; therefore, the variant in this gene was also classified as a variant of unknown significance. The other missense variants, in genes not previously associated with a neurodevelopmental disorder, were also all considered to be of unknown significance. Recurrence, segregation information, and/or functional evidence would be required to classify them otherwise. No structural variants affecting an exonic region of an intolerant gene were identified.

Turning to the ten cases with genome sequencing data of both parents available, a total of six rare *de novo* nonsynonymous variants were identified after filtering for minor allele frequency in public databases and for expression of the transcript in the (developing) brain (Table 3). Most of the identified *de novo* variants are predicted to be tolerated by the encoded protein (SIFT or PolyPhen indicating a tolerated/benign variant and/or REVEL < 0.25) or are located in a (region of a) gene tolerant to missense variants (MIS_Z < 3 or the local missense tolerance score indicating a tolerant region), and we therefore classified them as variants of unknown significance. Only one of the *de novo* variants is predicted to be damaging and is located in an intolerant gene: the stop-gain in *KDR* (c.C823T; p.R275X) in proband 14 (Figure 1(f)). KDR is a growth factor receptor tyrosine kinase that acts as a cell-surface receptor for vascular endothelial growth factor [68]. Sequencing studies in large developmental disorder cohorts previously reported *de novo* pLoF variants in *KDR* in one proband with a neurodevelopmental disorder [69] and in another with autism spectrum disorder, as well as a *de novo* missense variant in a case with autism spectrum disorder [70]. However, no statistically significant enrichment of (likely) pathogenic variants has yet been identified to prove a causal role of *KDR*. Recently, pLoF variants in *KDR* were associated with pulmonary arterial hypertension in a large cohort [71] and in two families [72] and with a congenital heart defect called tetralogy of Fallot [73]. Due to these conflicting reports, the *KDR* variant was classified as being of unknown significance.

## 4. Discussion

Here, we used genome sequencing to study the genetic underpinnings of idiopathic speech delay. We included 23 children with speech delay and also sequenced the genomes of both parents of ten of the probands to allow for the investigation of *de novo* variants. We only included children that had a diagnosis of speech delay without signs of intellectual disability, to avoid studying neurodevelopmental disorders with a broader phenotypic spectrum, which is common practice and has already led to the identification of many genes with causal variants [69]. In our idiopathic speech delay cohort, we identified a pathogenic frameshift variant in *SETD1A* and likely pathogenic variants in *SPTBN1* and *ARF3*. In the remaining 20 probands, multiple rare pLoF and likely deleterious missense variants were identified that might play causal roles in the observed speech delay, but that require additional evidence to be formally classified as pathogenic.

To our knowledge, this study provides the first case of a *SETD1A* disorder where only speech delay is the symptom, in the absence of other syndromic features. Prior cases have been reported with developmental delay, intellectual disability, subtle facial dysmorphisms, behavioural problems, early-onset epilepsy, schizophrenia, and/or CAS [13, 14, 17, 47, 74, 75]. Notably, speech or language delays, although typically not well defined, have been observed in the majority of reported *SETD1A* disorder cases, but never as the sole symptom. The identified pathogenic *SETD1A* variant is a deletion leading to a frameshift and a truncated protein that lacks several key functional domains, including the highly conserved SET domain which is essential for histone 3 lysine 4 methylation [76]. The variant is predicted to lead to nonsense-mediated mRNA decay, leading to typical *SETD1A* haploinsufficiency. It is therefore unlikely that this variant has reduced penetrance due to a mild effect on the protein. Moreover, the identical variant was independently reported to be pathogenic in a case of *SETD1A* haploinsufficiency disorder with broader and more severe symptoms including intellectual disability, global developmental delay, speech delay, and autism spectrum disorder [17]. It is likely that additional genetic, environmental, and/or stochastic factors modify the effects of pathogenic *SETD1A* variants, leading to the relatively speech-specific phenotype observed in proband 01. Variable expressivity and pleiotropy have previously been shown for *SETD1A,* as well as a set of genes implicated in monogenic syndromes, for which neurodevelopmental disorders and schizophrenia are part of the clinical spectrum [17, 47, 75]. Our results indicate that the full clinical spectrum associated with *SETD1A* haploinsufficiency also includes speech delay in the absence of intellectual disability or global developmental delay.

Remarkably, in recent studies, *de novo* pLoF variants in *SETD1A* have been identified twice in children ascertained based on their speech disorder, despite the relatively modest size of the cohorts being screened [13, 14]. In cohorts with people with speech disorders, *SETD1A* haploinsufficiency therefore seems rather frequent (2 out of 123 people with CAS and 1 out of 23 people with speech delay) [13–15], compared to cohorts with people with schizophrenia (10 out of 7,776) or a neurodevelopmental disorder (4 out of 11,110) [47]. The full extent to which CAS and idiopathic speech delay are caused by pathogenic *SETD1A* variants has yet to be revealed, because cases with mild symptoms have likely been undersampled in the clinical populations from which most published cases have been identified. This is also evident from, for example, the enrichment of penetrance-increasing cis-regulatory variants [77] and risk-increasing common genetic variation [78] in probands with a monogenic disorder in clinical cohorts. Still, very few pLoF variants have been found in *SETD1A* in large population databases with sequencing data like gnomAD [33]. Therefore, it is likely that *SETD1A* disorder generally does not go unobserved and undiagnosed. Unbiased genotype-to-phenotype studies are required to identify the full spectrum of phenotypes associated with *SETD1A* haploinsufficiency. Given the high yield of rare high-penetrance variants, our results indicate that probands with profound developmental speech disorders such as CAS or severe speech delay should be considered for genetic screening.

In our speech delay cohort, likely pathogenic variants were also identified in *SPTBN1* and *ARF3*. Both genes have already been associated with a neurodevelopmental disorder, in which speech delay has been reported in the majority of cases but never as the sole symptom [50–52, 56]. In *ARF3*-related disorder, speech is completely absent in multiple cases. The probands described here likely represent very mild cases of the clinical spectrum of the disorders associated with these genes. The variants reported here in *SPTBN1* and *ARF3* are missense variants, and future functional assays in animal and cellular systems may help clarify further the effects of the variants on the encoded proteins. Given several lines of evidence (the variants are novel (i.e., never previously observed in DNA sequencing data from large numbers of healthy individuals), predicted to be damaging, and affect a key amino acid in a highly conserved functional domain), it is likely that both variants are pathogenic. For 20 probands, no pathogenic or likely pathogenic variants were identified. Where the trio approach [13, 14] and singleton approach [13, 15] were previously successful in identifying high-confidence variants for CAS in 26-42% of cases, the yield is much lower (13%) for probands with speech delay, and no additional benefit was obtained from the trio approach taken for half of the cohort.

Genome sequencing is a powerful method to identify genetic variants within and outside coding regions with high quality. In the present study, by including only probands without signs of intellectual disability or other syndromic features, we were able to search for genes implicated in speech delay against a background of relatively preserved general cognitive function. These analyses are however limited by our sample size, reducing the chance of identifying multiple pathogenic variants in the same gene, which could help provide stronger evidence for causal relations. As this represents the first next-generation sequencing analysis to focus on idiopathic speech delay, we had to rely on associations with other speech or neurodevelopmental disorders for validating pathogenic roles. Our strict inclusion criteria may have therefore limited the number of (likely) pathogenic variants we could successfully identify. Sequencing DNA of patients and their healthy parents has been a highly successful approach in identifying pathogenic *de novo* variants for many neurodevelopmental disorders, including severe speech phenotypes like CAS, but may be less suitable for milder forms of speech impairment. Finally, we note that some of the variants that we identified in the current study might in future be classified as (likely) pathogenic, when more evidence for causal roles of the relevant genes is gathered from larger sequencing datasets.

## Figures and Tables

**Table 1:** Rare probable loss-of-function variants in intolerant genes identified in 13 singleton cases with speech delay.

Proband	Chr	Base (GRCh37)	Base (GRCh38)	Gene	Transcript	Variant effect	cDNA change	Protein change	gnomAD MAF	pLI	Phenotype previously associated with heterozygous pLoF variants	Classification
01	16	30991037	30979716	*SETD1A*	NM_014712	Frameshift	c.3930_3940del CCCTGCGCCAG	p.P1313Afs^∗^17	0	1.00	Neurodevelopmental disorder and schizophrenia [47], childhood apraxia of speech [13, 14]	Pathogenic
02	2	242122172	241182757	*PPP1R7*	NM_002712	Frameshift	c.1017delC	p.R340Gfs^∗^111	0	0.99	—	VUS
04	5	145859585	146480022	*TCERG1*	NM_006706	Splicing	c.1820-3_1822delTAGTTA	?	2.2 × 10^−5^	1.00	—	VUS
06	16	67580073	67546170	*RIPOR1*	NM_024519	Stop-gain	c.C3501G	p.Y1167X	0	0.95	—	VUS
	17	38572722	40416470	*TOP2A*	NM_001067	Frameshift	c.220delG	p.E74Kfs^∗^16	0	0.99	—	VUS

Chr: chromosome; MAF: minor allele frequency; pLI: probability of being loss-of-function intolerant; VUS: variant of unknown significance.

**Table 2:** Rare likely deleterious missense variants in intolerant genes identified in 13 singleton cases with speech delay.

Proband	Chr	Base (GRCh37)	Base (GRCh38)	Gene	Transcript	cDNA change	Protein change	gnomAD MAF	MIS_Z	pLI	Local missense intolerance	SIFT	PolyPhen	REVEL	GERP	Phenotype previously associated with heterozygous variants	Classification
01	1	53742664	53276992	*LRP8*	NM_001018054	c.G583A	p.G195S	2.5 × 10^−5^	2.77	1.00	Highly intolerant	D	P	0.669	2.91	—	VUS

02	1	10318654	10258596	*KIF1B*	NM_015074	c.A287G	p.Y96C	4.0 × 10^−6^	3.6	1.0	Intolerant	D	D	0.95	5.7	Charcot-Marie-Tooth neuropathy	VUS

03	4	140282956	139361802	*NAA15*	NM_057175	c.G1618T	p.D540Y	8.0 × 10^−6^	3.8	1.0	Intolerant	D	D	0.54	6.0	Intellectual disability, delayed speech and motor milestones, and autism spectrum disorder [53, 54]	VUS

04	2	54845255	54618118	*SPTBN1*	NM_003128	c.G688A	p.A230T	0	4.5	1.0	Intolerant	D	D	0.86	5.6	Intellectual disability, delayed speech, autistic features, and seizures [50, 51]	Likely pathogenic
	6	165863831	165450343	*PDE10A*	NM_001130690	c.A245C	p.Q82P	0	3.8	1.0	Intolerant	D	D	0.53	4.6	Striatal degeneration [61]	VUS

05	7	103124179	103483732	*RELN*	NM_005045	c.G10102A	p.G3368R	5.7 × 10^−5^	1.14	1.00	Intolerant	D	D	0.703	5.72	Autism [62], epilepsy [63], neurodevelopmental disorders [64], lissencephaly [65]	VUS/likely benign^∗^
	10	27405184	27116255	*YME1L1*	NM_001253866	c.G1711A	p.E571K	0	2.06	0.99	Intolerant	D	D	0.943	5.43	—	VUS
	12	49333815	48940032	*ARF3*	NM_001659	c.G224C	p.R75P	0	3.01	0.62	Highly intolerant	D	D	0.881	3.83	Neurodevelopmental disorder with brain and skeletal abnormalities [52]	Likely pathogenic
	14	35240770	34771564	*BAZ1A*	NM_182648	c.C3152T	p.A1051V	4.0 × 10^−6^	2.69	1.00	Intolerant	T	D	0.636	5.66	—	VUS

06	19	42777266	42273114	*CIC*	NM_001304815	c.G1331T	p.C444F	0	1.53	1.00	NA	NA	NA	NA	4.7	Intellectual disability, autism spectrum disorder, and ADHD [60]	VUS

07	9	140069703	137175251	ANAPC2	NM_013366	c.G2242A	p.E748K	4.0 × 10^−6^	2.43	1.00	Intolerant	D	D	0.506	4.32	—	VUS
	19	41183304	40677399	NUMBL	NM_001289979	c.T440C	p.V147A	0	3.18	1.00	Intolerant	D	D	0.596	5.31	—	VUS

08	5	45695972	45695870	*HCN1*	NM_021072	c.223_224ins GCGGCGGCG	p.G74_E75 insGGG	7.2 × 10^−5^	3.72	1.0	Intolerant	NA	NA	NA	NA	Infantile epileptic encephalopathy [66]	Likely benign^∗^
	12	122626291	122141744	*MLXIP*	NM_014938	c.C2692G	p.P898A	0	1.87	0.98	Intolerant	D	D	0.72	5.5	—	VUS

09	9	130422360	127660081	*STXBP1*	NM_001032221	c.C298T	p.R100W	7.1 × 10^−6^	4.3	1.0	Intolerant	D	D	0.70	4.6	STXBP1-related disorders including neurodevelopmental delay and seizures [67]	Likely pathogenic/VUS^∗^
	10	75557014	73797256	*ZSWIM8*	NM_001242487	c.3403_3405del	p.K1137del	1.8 × 10^−5^	5.53	1.00	Intolerant	NA	NA	NA	NA	—	VUS

10	11	120312519	120441810	*ARHGEF12*	NM_001198665	c.C1139A	p.A380E	0	3.3	1.0	Highly intolerant	D	D	0.82	5.5	—	VUS
	20	50307357	51690818	*ATP9A*	NM_006045	c.A644G	p.D215G	2.1 × 10^−5^	4.2	1.0	Intolerant	D	D	0.79	5.3	—	VUS

Rare, likely deleterious missense variants located in brain-expressed transcripts intolerant to missense variation are listed. Chr: chromosome; MAF: minor allele frequency; MIS_Z: *Z*-score for missense constraint; pLI: probability of being loss-of-function intolerant; SIFT: Sorting Intolerant From Tolerant; PolyPhen: Polymorphism Phenotyping; REVEL: rare exome variant ensemble learner; GERP: Genomic Evolutionary Rate Profiling; NA: not available/not applicable; T: tolerated/benign; P: possibly damaging; D: deleterious; VUS: variant of unknown significance. ^∗^Classification from ClinVar [44].

**Table 3:** *De novo* exonic variants in the 10 speech delay proband-parent trios.

Proband	Chr	Base (GRCh37)	Base (GRCh38)	Gene	Transcript	Variant effect	cDNA change	Protein change	gnomAD MAF	pLI	MIS_Z	Local missense tolerance	SIFT	PolyPhen	REVEL	GERP	Classification
14	4	55979624	55113457	*KDR*	NM_002253	Stop-gain	c.C823T	p.R275X	0	1.00	NA	NA	NA	NA	NA	5.5	VUS
15	2	101620700	101004238	*RPL31*	NM_000993	Missense	c.G188A	p.R63H	4.0 × 10^−6^	NA	1.6	Intolerant	T	T	0.77	4.2	VUS
16	3	52556182	52522166	*STAB1*	NM_015136	Missense	c.A6401G	p.N2134S	0	NA	1.1	Slightly tolerant	D	P	0.44	5.6	VUS
17	11	18723515	18701968	*TMEM86A*	NM_153347	Missense	c.C682T	p.R228W	2.0 × 10^−5^	NA	0.9	Slightly tolerant	D	D	0.17	5.6	VUS
18	16	77325369	77291472	*ADAMTS18*	NM_199355	Missense	c.G3196C	p.A1066P	0	NA	-3.5	Slightly intolerant	D	P	0.23	1.5	VUS
19	19	17631811	17521002	*PGLS*	NM_012088	Missense	c.C698A	p.T233N	0	NA	0.2	Intolerant	T	T	0.09	5.3	VUS

All rare nonsynonymous *de novo* variants in brain-expressed transcripts are listed; the variants are not filtered for (local) gene intolerance and predicted functional impact. Chr: chromosome; MAF: minor allele frequency; pLI: probability of being loss-of-function intolerant; MIS_Z: *Z*-score for missense constraint; SIFT: Sorting Intolerant From Tolerant; PolyPhen: Polymorphism Phenotyping; REVEL: rare exome variant ensemble learner; GERP: Genomic Evolutionary Rate Profiling; NA: not available/not applicable; T: tolerated/benign; P: possibly damaging; D: deleterious; VUS: variant of unknown significance.

## Data Availability

The primary data for the study have been deposited at the MPI for Psycholinguistics Archive (https://archive.mpi.nl/mpi/), a public data archive hosted by the Max Planck Institute for Psycholinguistics. Data are accessible with the persistent identifier (https://hdl.handle.net/1839/f2544d71-3ca6-4239-83c8-66d81f71e6c4). Access can be granted upon request.

## Supplementary Materials

Supplementary Materials Supplemental Table 1 contains an overview of clinical characteristics of the 23 probands included in the genome sequencing study.
