# Supplementary material for: Genome Sequencing of Idiopathic Speech Delay
Source: Hum Mutat. 2024 Mar 28;2024:9692863. doi: 10.1155/2024/9692863 (PMC11918988; doi:10.1155/2024/9692863)
Supplement: Supplementary Materials — Supplemental Table 1 contains an overview of clinical characteristics of the 23 probands included in the genome sequencing study. [file 9692863.f1.docx]

**Supplemental Table 1: Overview of clinical characteristics of the 23 probands included in the genome sequencing study**

| Proband | Age (years) | Sex | General Ability^a,b^ | Speech delay | Late onset language use | Expressive and receptive oral language^b,c^ | Articulation^b,d^ | Consonants correct^e^ | Vowels correct^e^ | Age beginning speech/language therapy (years;months) | Gross and/or fine motor impairment^f^ | Other |
| --- | --- | --- | --- | --- | --- | --- | --- | --- | --- | --- | --- | --- |
| 01 | 4 | F | 96 | + |  | 91 | 55 | -5 | -5 | 2 | + |  |
| 02 | 8 | M | 108 | + |  | 113 | 87 | -3.56 | -0.68 | 6 |  |  |
| 03 | 9 | M | 83 | + | + | 72 | <40 | -4.05 | 0.18 | 2;6 | + | C-section at 37 weeks. Chewing problems |
| 04 | 6 | F | 96 | + | NA | 101 | 74 | -5 | -2.49 | 5 |  |  |
| 05 | 5 | F | 96 | + | + | 90 | 59 | -3.45 | -4.44 | 2;6 |  |  |
| 06 | 4 | M | 99 | + |  | 103 | 81 | -2.68 | -4.7 | 2;6 |  | Restrictive/tight frenum affecting speech and feeding; recurrent ear infections |
| 07 | 7 | M | NA | + |  | 108 | <40 | -5 | -3.77 | 1;6 | + | Learning disability, ADHD |
| 08 | 5 | M | 98 | + | + | 90 | 93 | -1.44 | -3.46 | 2;6 | + | C-section at 34.5 weeks. Febral seizures at 11 months. |
| 09 | 4 | M | 97 | + | NA | 94 | 79 | -2.37 | -5 | 2 |  |  |
| 10 | 3 | F | 86 | + |  | 102 | 79 | -3.27 | -4.72 | NA |  |  |
| 10 | 7 | M | 96 | + |  | 90 | 95 | -1.66 | -2.78 | 3 |  |  |
| 11 | 8 | M | 93 | + | NA | 101 | 83 | -0.86 | -5 | NA |  |  |
| 12 | 6 | M | 97 | + |  | 101 | 94 | -3.08 | -0.19 | 5 | + | C-section due to partially separated placenta |
| 14 | 7 | M | 111 | + | NA | 111 | 99 | -1.17 | -2.7 | NA |  |  |
| 15 | 6 | M | 106 | + |  | 103 | 96 | -0.86 | -2.64 | 5 |  |  |
| 16 | 3 | M | 99 | + |  | 102 | 99 | -2.03 | -5 | 2 |  |  |
| 17 | 5 | M | 107 | + |  | 104 | 106 | -1.05 | -5 | 4 |  | Drooling problems from 0 to 4 years of age |
| 18 | 7 | M | 99 | + |  | 88 | 95 | -2.74 | -5 | 4 |  |  |
| 19 | 5 | M | NA | + |  | 119 | 102 | -0.37 | -5 | NA |  | Chronic ear infections, 2 sets of tubes |
| 20 | 5 | M | 111 | + | + | 53 | 107 | -0.84 | -3.09 | 2 |  | Oral pharyngeal dysphasia |
| 21 | 3 | M | 100 | + | + | 98 | 93 | -1.27 | -2.06 | 2;3 |  |  |
| 22 | 5 | M | 108 | + |  | 109 | 91 | -0.93 | -5 | 4 |  |  |
| 23 | 6 | M | 119 | + |  | 102 | 80 | -0.95 | -3.68 | 4 |  |  |

Plus-sign ('+') indicates impairment, blank cells indicate negative history or performance within normal limits. ^a^Detroit Test of Learning Aptitude-Primary 3^rd^ edition; **^b^**Standard score. ^c^Oral and Written Language Scales 2^nd^ edition. ^d^Goldman-Fristoe Test of Articulation. ^e^Z-score. ^f^Based on parent report or history of physical or occupational therapy. NA: not available.
